# Physicochemical Properties of Ion Pairs of Biological Macromolecules

**DOI:** 10.3390/biom5042435

**Published:** 2015-09-30

**Authors:** Junji Iwahara, Alexandre Esadze, Levani Zandarashvili

**Affiliations:** Department of Biochemistry & Molecular Biology, Sealy Center for Structural Biology and Molecular Biophysics, University of Texas Medical Branch, Galveston, TX 77555, USA

**Keywords:** dynamics, electrostatic interactions, electrostriction, entropy, free energy, ion pairs, nucleic acids, proteins, salt bridges

## Abstract

Ion pairs (also known as salt bridges) of electrostatically interacting cationic and anionic moieties are important for proteins and nucleic acids to perform their function. Although numerous three-dimensional structures show ion pairs at functionally important sites of biological macromolecules and their complexes, the physicochemical properties of the ion pairs are not well understood. Crystal structures typically show a single state for each ion pair. However, recent studies have revealed the dynamic nature of the ion pairs of the biological macromolecules. Biomolecular ion pairs undergo dynamic transitions between distinct states in which the charged moieties are either in direct contact or separated by water. This dynamic behavior is reasonable in light of the fundamental concepts that were established for small ions over the last century. In this review, we introduce the physicochemical concepts relevant to the ion pairs and provide an overview of the recent advancement in biophysical research on the ion pairs of biological macromolecules.

## 1. Introduction

Ion pairing is one of the most fundamental atomic interactions in chemistry and biology. Ion pairs of electrostatically interacting cationic and anionic moieties are important for proteins and nucleic acids to perform their function. The importance of the ion pairs (also known as salt bridges) in protein function is evident from numerous three-dimensional structures of protein–protein and protein–DNA/RNA complexes [1,2,3]. Crucial intermolecular ion pairs are often found in the structures of protein–drug complexes [4,5,6,7,8], suggesting that deeper knowledge of ion pairs can improve drug design.

Despite the fundamental importance of biomolecular ion pairs, it seems that their physicochemical properties are not well known in the molecular biology, structural biology, biophysics, and biochemistry research communities. In these fields, even major textbooks that were written or revised in the 21st century provide only simplistic descriptions of ion pairs as short-range electrostatic interactions, and these resources do not adequately cover other fundamental issues of ion pairs in biomolecular systems. Recent research has demonstrated various aspects of ion pairs that are obviously very important, not only for our understanding of protein and nucleic acid functions, but also for protein engineering and drug development.

The objective of this review is to introduce fundamental concepts, methodology, and recent advancements in research on biological ion pairs. We assume a broad readership from the fields of molecular biology, structural biology, biophysics, and biochemistry. In Section 2, we introduce fundamental physicochemical concepts, most of which were originally established for small molecule ion pairs. In Section 3, we describe recent advances in the experimental studies of the ion-pair dynamics in biological macromolecules. In Section 4, we introduce the methods for the thermodynamic investigations of the biomolecular ion pairs. In Section 5, we discuss some issues that need to be addressed in the future.

## 2. Fundamental Concepts on Ion Pairs

In this section, we introduce fundamental concepts related to ion pairs and interacting water. Most of these concepts were established for ion pairs of small molecule solutes in water. To understand ion pairs in biological systems, it should be noted that the role of water is extremely important.

### 2.1. Contact Ion Pair (CIP) and Solvent-Separated Ion Pair (SIP)

**Figure 1:**
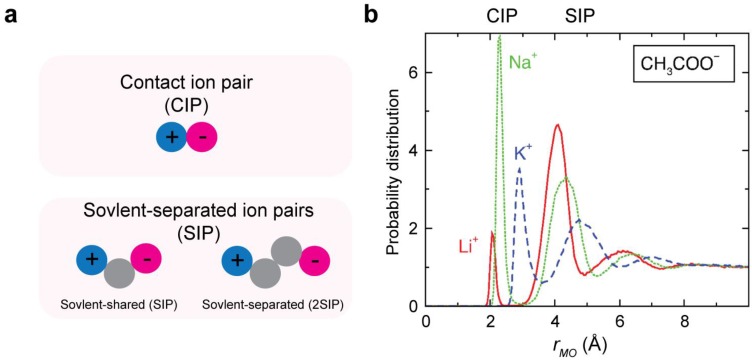
Contact ion pair (CIP) and solvent-separated ion pair (SIP). (**a**) Schematic of the CIP and SIP states. In some studies, SIP is subcategorized into solvent-shared ion pairs and solvent-separated ion pairs. (**b**) Radial distribution functions (RDFs) for the ion pairs of the alkali-acetate ion pairs. Panel b was adapted from Hess and van der Vegt [19].

One can distinguish two major states in which interacting cation and anion are either in direct contact or intervened by water molecule(s) (Figure 1). These states are called contact ion pair (CIP) and solvent-separated ion pair (SIP) states [9,10,11,12,13]. In some of the literature, ion pairs with a single water molecule intervening the ions are also called “solvent-shared” ion pairs. In this review, we refer to any ion pairs that are separated by a single or by multiple water molecules as SIP. Whether an ion pair prefers a CIP or SIP state depends on the type of the involved ions [9,10,11,12,13]. For example, LiF forms a stable CIP in water, whereas a LiI pair prefers a SIP state [9,11]. The preference between the CIP and SIP states of small ion pairs generally obeys the law of matching water affinity [9,10], as explained in Section 2.2. The CIP–SIP equilibria are often represented by a radial distribution function (RDF), which provides a probability distribution as a function of the interionic distance.

It is important to note that ion pairs are typically in dynamic equilibria between these CIP and SIP states [14,15,16,17,18]. For small molecule ion pairs, the dynamic transitions between the CIP and SIP states have been studied with time-resolved absorption spectroscopy since the 1980s, as described in Section 3.1. For biological macromolecules, the experimental studies of the CIP–SIP transitions remain very rare due to practical difficulties. Mainly in the 21st century, NMR methods for investigating the dynamics of charged moieties of biological macromolecules were developed, which enabled some investigations on the ion-pair dynamics for proteins and nucleic acids, as described in Section 3.2. Although the crystal structures of biological macromolecules show typically either a CIP or SIP state for each ion pair, dynamic equilibria involving the CIP and SIP states should exist in solution.

### 2.2. Electrostriction of Water Molecules by Ions

**Figure 2:**
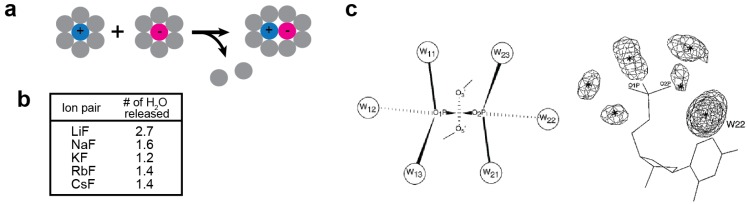
Electrostriction of water molecules around ions. (**a**) Schematic of the ion pair formation and consequent release of water molecules. (**b**) Numbers of released water molecules for alkali-fluoride ion pairs [22]. (**c**) Six hydration sites around DNA phosphate group. On the right is a probability density map of hydration water from the statistical analysis of the crystal structures. Panel c was adapted from Schneider and Berman [23] with permission from Elsevier.

Ions in solution create strong electric fields that strongly impact the dipole of the surrounding water. As a result, the ions rearrange the nearby water molecules, bind to them, and restrict their freedom of motion. This effect, which is called solvent binding or electrostriction, leads to a reduction in entropy [20]. Ion pair formation considerably diminishes the strong electric fields of the individual ions, and thereby loosens the electrostriction of water. Consequently, as schematically shown in Figure 2a, the total number of restricted water molecules decreases, and some of the water molecules are released upon ion-pair formation (“desolvation”). For example, experimentally determined numbers of the water molecules released by alkali-fluoride ion pairs are given in Figure 2b.

The release of water molecules upon ion-pair formation results in an increase in the entropy of the system. For small ion pairs, these entropic effects have been well studied experimentally [21,22]. In fact, for many cases, the ion-pair formation is entropy-driven by the release of water molecules. The change in entropy due to desolvation usually is smaller for SIP than for the CIP, because a smaller number of water molecules are released upon the formation of SIP.

Electrostriction of water molecules occurs for ionic moieties of biological macromolecules as well. For example, statistical analysis of high-resolution crystal structures showed six hydration sites around each phosphate group of DNA (Figure 2c) [23] and four hydration sites around each carboxylate group of the aspartate or glutamate side chain of proteins [24,25].

### 2.3. Kosmotropic and Chaotropic Ions

Ions can be categorized into kosmotropes (“order maker”) and chaotropes (“order breaker”) according to their influence on the hydrogen-bonding network of water. Kosmotropic ions cause strong electrostatic ordering of nearby water molecules. Chaotropic ions only weakly interact with water, and their interaction is weaker than the water–water interaction. In 1929, Jones and Dole studied the influence of ions on water viscosity and found the following relationship [26]:

(1) $$\eta /\eta_{0}=1+A\sqrt{c}+Bc$$

where η/η_0_ is a relative viscosity of the solvent to pure water; *c* is a salt concentration; *A* is an electrostatic parameter identical for all ions; and *B* is a parameter called the “Jones-Dole coefficient”. A positive *B* coefficient indicates that the ions increase the water viscosity by stabilizing the hydrogen-bonding network of water, and a negative *B* coefficient indicates that the ions reduce the water viscosity, destabilizing the water-water interactions. Jones-Dole *B* coefficients are positive for kosmotropic ions and negative for chaotropic ions. Kosmotropic ions are typically those with a small radius and a high charge density, whereas chaotropic ions are those with a large radius and a low charge density.

Although kosmotropes and chaotropes were concepts defined originally for small molecule solutes, these terms are also used for charged moieties in biological macromolecules [9,10]. In nucleic acids, negatively charged phosphate groups are considered kosmotropic anions. In proteins, positively charged moieties of Lys and Arg side chains are considered to be chaotropic, and negatively charged moieties of Asp and Glu side chains to be kosmotropic. These classifications are based on the properties of the corresponding small ions [9,10].

### 2.4. Collins’s Law of Matching Water Affinity

**Figure 3:**
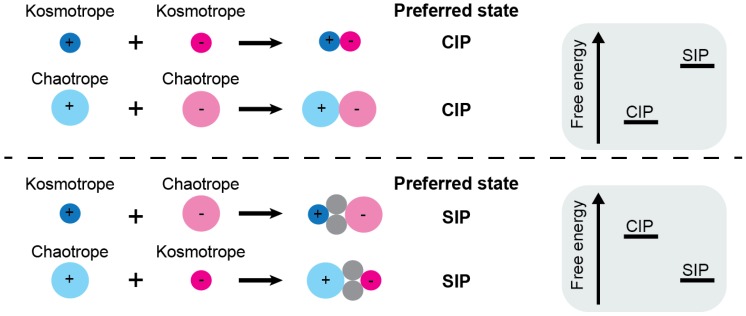
Collins’s law of matching water affinity. Kosmotrope–kosmotrope ion pairs and chaotrope–chaotrope ion pairs prefer the CIP state (*i.e.*, lower free energy for CIP, as observed for LiF in Figure 4b). Kosmotrope–chaotrope ion pairs prefer the SIP state (*i.e.*, lower free energy for SIP, as observed for CsF in Figure 4b).

With regard to the propensities of CIP and SIP states for ion pairs, Collins proposed an empirical rule [9], which is often referred to as the law of matching water affinity. According to this law, the CIP state is preferred if the cation and anion have similar affinities for water. Kosmotropic ions possess a stronger affinity for water than chaotropic ions do. Thus, kosmotrope–kosmotrope and chaotrope-chaotrope ion pairs prefer the CIP state (Figure 3). Kosmotrope–kosmotrope ion pairs prefer the CIP state because their direct ionic interaction is stronger than the interactions between the water and each kosmotropic ion. Chaotrope–chaotrope ion pairs also prefer the CIP state because the water molecules released from the CIP will create additional water–water interactions that are more stable than the water–chaotrope interactions. Kosmotrope–chaotrope ion pairs prefer the SIP state because the water–water interactions and water–chaotrope interactions are weaker than the water–kosmotrope interactions. Collins deduced this qualitative law from various data on alkali-halide salts, including Jones-Dole coefficients, solubility, and standard heats of solution [9]. The validity of this empirical law was confirmed for alkali–halide and alkali–acetate ion pairs through computational studies [11,19,27,28,29,30].

Despite its success for small ion pairs, Collins’s law may not necessarily be applicable to ion pairs of cationic and anionic moieties of biological macromolecules. For example, consider the intermolecular ion pairs of protein lysine (Lys)/arginine (Arg) side-chain cations and DNA phosphate anions. According to Collins’s law, the preferred state of these intermolecular ion pairs should be SIP because the cationic moieties of the Lys and Arg side chains are chaotropic and the DNA phosphate groups are kosmotropic. Despite this prediction, the crystal structures of many protein–DNA/RNA complexes show a preference for the CIP state. Furthermore, recent solution NMR studies on ion-pair dynamics have suggested that side-chain NH_3_^+^-phosphate ion pairs at the molecular interface of protein–DNA complexes prefer the CIP state [31,32,33]. This could be due to some factors specific to macromolecular ion pairs (e.g., restriction by covalent bonds, complex energy surface).

**Figure 4:**
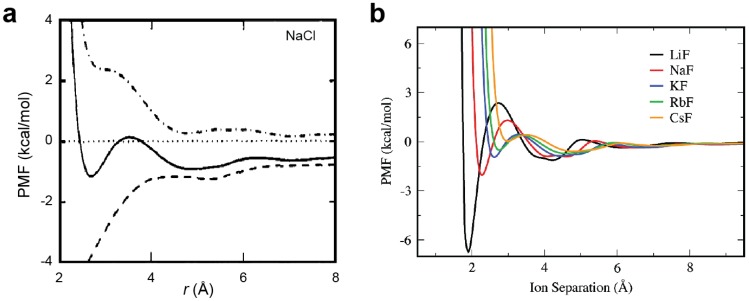
Potentials of mean force (PMFs) for alkali-halide ion pairs in water. (**a**) PMF for Na^+^-Cl^−^ ion pair in water. The solid line represents free energy (Δ*G*); the dot-dashed line represents the enthalpic term (Δ*H*); and the dashed line represents the entropic term (-*T*Δ*S*). Adapted from Pettitt and Rossky [34] with permission from AIP Publishing. (**b**) PMFs for alkali-F^−^ ion pairs. Adapted from Fennell *et al.* [11] with permission from American Chemical Society.

### 2.5. Potentials of Mean Force (PMFs) for Ion Pairs

Potentials of mean force (PMFs) for ion pairs represent the free-energy landscape, which is typically given as a function of the interionic distance in a particular solvent. PMFs for ion pairs are calculated theoretically [34] or via molecular dynamics (MD) or Monte Carlo (MC) simulations [11,35,36,37,38,39,40]. PMFs are useful for understanding free energy differences between CIP and SIP or completely dissociated states as well as the energy barriers between the states. PMFs of ion pairs typically show multiple minima. The first minimum with the shortest interionic distance corresponds to the CIP state. The second minimum corresponds to the SIP state. Based on the free energies of the CIP and SIP states, one can estimate the relative populations of the CIP and SIP states. The peak between the CIP and SIP minima corresponds to the energy barrier for the CIP–SIP transitions, provided that the PMFs represent the reaction coordinates. If the energy barrier is higher, the CIP–SIP transitions should be slower. Thus, PMFs are very useful in understanding the physicochemical properties of ion pairs.

Using MD simulations, Fennell *et al.* [11] obtained PMFs for the full set of alkali-halide ion pairs and examined the validity of Collins’s law. They applied different force-field parameter sets for the calculations and found the same trends in the PMFs, but they obtained different free energies of ion pairs depending on the force-field parameters. Nevertheless, each dataset appeared to be qualitatively consistent with Collins’s law (Figure 4). For example, the PMF for the Li^+^-F^−^ (kosmotrope–kosmotrope) ion pair shows a CIP free energy lower than the SIP free energy (black), which indicates a preference for CIP. In contrast, the PMF for the Cs^+^-F^−^ (chaotrope–kosmotrope) ion pair shows a CIP free energy higher than the SIP free energy (yellow), which indicates a preference for SIP.

**Figure 5:**
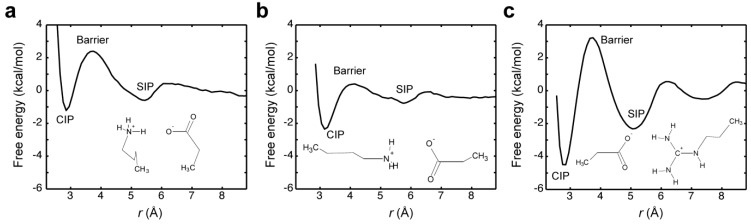
PMFs for the ion pairs of free amino-acid side chains [38]. (**a**) Lys^+^-Glu^−^ ion pair in a side-by-side orientation. (**b**) Lys^+^-Glu^−^ ion pair in a head-to-head orientation. (**c**) Arg^+^-Glu^−^ ion pairs in a head-to-head orientation. Data courtesy of Prof. Themis Lazaridis (City University of New York).

Masunov and Lazaridis calculated PMFs for ion pairs of free amino-acid side chains using MD simulations [38]. Figure 5 shows the PMFs for free Lys^+^-Glu^−^ and Arg^+^-Glu^−^ ion pairs. Their PMFs depend strongly on their relative orientation: with a side-by-side orientation, the free energy difference between the CIP and SIP states (Δ*G*°_CIP_–_SIP_) is only ~0.3 kcal/mol (Figure 5a), but with a head-to-head orientation, the free energy of the CIP state of Lys^+^-Glu^−^ ion pair is lower than that of its SIP state by ~1.3 kcal/mol (Figure 5b). When the PMFs for Arg^+^-Glu^−^ and Lys^+^-Glu^−^ ion pairs in head-to-head orientation are compared, their free-energy difference between the CIP and SIP states are comparable; however, the energy barrier for the transition from the CIP to SIP state (Δ*G*^‡^_CIP_–_SIP_) is substantially higher for the Arg^+^-Glu^−^ ion pair (compare Figure 5b,c). This result predicts that the transitions between the CIP and SIP states for an Arg^+^-Glu^−^ ion pair should take more time than those for a Lys^+^-Glu^−^ ion pair in the same orientation. Though this is an interesting possibility, it remains to be tested experimentally.

**Figure 6:**
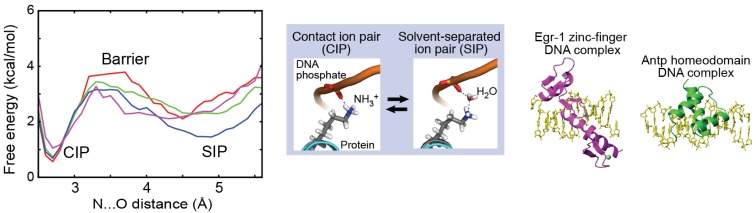
PMFs for the intermolecular ion pairs of the protein side-chain NH_3_^+^ and DNA phosphate groups in the Antp homeodoman–DNA complex and the Egr-1 zinc-finger–DNA complex. Red line presents the PMFs for Lys46 (Antp); green, Lys55 (Antp); blue, Lys57 (Antp); and magenta, Lys79 (Egr-1). Adapted from Chen *et al.* [32].

Recently, using 0.6-μs MD simulations, Chen *et al.* obtained PMFs for the intermolecular ion pairs of protein side-chain NH_3_^+^ and DNA phosphate groups in the Antp homeodomain–DNA complex and the Egr-1 zinc-finger–DNA complex [32]. Figure 6 shows the PMFs for the intermolecular ion pairs of the protein side-chain NH_3_^+^ and DNA phosphate groups. For the intermolecular ion pairs whose CIP states were experimentally detected, the free energy differences, Δ*G_o_*(CIP → SIP), were determined to be 0.8–1.6 kcal/mol at the standard temperature. The energy barriers, Δ*G*^‡^(CIP → SIP), for the escape from the CIP state were determined to be 2.2–3.2 kcal/mol, which are qualitatively consistent with the mean lifetimes of the CIP states in the simulations. The variation in the energetics among the different residues is most likely related to differences in the local environments.

### 2.6. pK_a_ Shift

The parameter p*K_a_* for a titratable group is a useful measure of the ionization equilibrium and corresponds to the pH value at which the populations of the protonated and deprotonated states become equal. The ion-pair formation of biological macromolecules typically causes shifts of the ionization equilibria of the titratable moieties. In fact, the catalytic residues of some enzymes form ion pairs and exhibit abnormal p*K_a_* values. For example, two catalytic side chains Lys167 and Lys201 of 2-deoxyribose-5-phosphate aldolase exhibit a p*K_a_* that is shifted to a neutral range, largely due to the ion pair formation with Asp102 and Asp16, facilitating direct contact of the two N_ζ_ atoms [41,42,43]. While ion-pair formation is an influential factor for ionization equilibrium, other factors could also strongly influence p*K_a_* [44,45]. For example, lysine side-chain amino groups buried in hydrophobic environment exhibit unusually low p*K_a_* (as low as 5.6) [46,47,48,49,50,51], thus increasing the population of their deprotonated state (*i.e.*, NH_2_), which is important for some enzymes [46,49]. There are some computational methods for the structure-based prediction of p*K_a_* values (e.g., reviewed in References [43,44,52]).

### 2.7. Polyelectrolyte Effects

Macromolecules with a large number of charged moieties attract many counterions through long-range electrostatic interactions and the population of counterions around the macromolecules can become substantially higher than the overall mean concentration in solution. Manning proposed a theory to describe this phenomenon for polyelectrolyte chains that can be represented as a linear array of point charges, with a finite local volume for condensation per point charge [53,54,55]. Manning defined a parameter *ξ*, which is useful in judging whether or not the counterion condensation occurs:

(2) $$\xi =\frac{l_{B}}{b}=\frac{e^{2}}{4\pi \varepsilon_{0}Dk_{B}Tb}$$

where *b* is the spacing between the charges along the axis; *l_B_*, the Bjerrum length characteristic of the solvent (*l_B_* = 7.1 Å for water at standard temperature); *e*, the elementary charge; *ε*_0_, the vacuum permittivity; *D*, the dielectric constant; *k*_B_, the Boltzmann constant; and *T*, the temperature. Counterion condensation occurs only when this parameter satisfies *ξ* > 1. Most proteins typically do not satisfy this condition, and counterion condensation thus does not occur around them. However, because *b* = 1.7 Å for B-form DNA (two phosphate groups per 3.4 Å), the condition of *ξ* > 1 is satisfied and counterion condensation does occur around DNA [15]. In fact, the counterion condensation around DNA was directly evidenced by solution X-ray scattering [56,57] and by atomic emission spectroscopy [58].

Manning also defined the local concentration [*M*^+^]_cond_ of condensed counterions to be:

(3) $${\left[M^{+}\right]}_{cond}=\frac{1}{8\pi \text{e}\xi b^{3}}\frac{1}{1000N_{A}}$$

where e is the base of natural logarithm and *N*_A_ is the Avogadro’s number. The factor of (1000*N*_A_)^−1^ is for units of mol/L (*i.e.*, M). This equation gives [*M*^+^]_cond_ = 1.2 M for B-form DNA [59]. It is important to note that [*M*^+^]_cond_ is independent of the total cation concentration.

**Figure 7:**
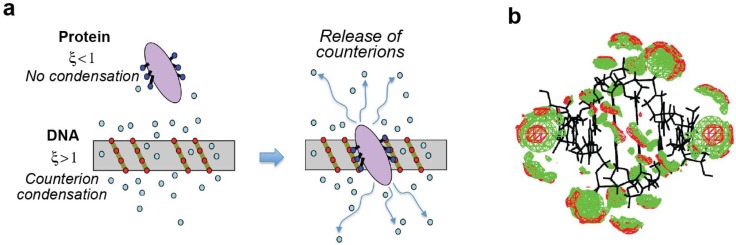
Polyelectrolyte effect of DNA. (**a**) Condensation of counterions around DNA and their release upon protein–DNA complex formation. Red circles represent the phosphate anions of DNA. Cyan circles represent the cations condensed around DNA. Blue circles on the protein represent the positively charged groups that form ion pairs with DNA. (**b**) Computed probability density maps of K^+^ (red) and Na^+^ (green) ions around B-form DNA at 0.1 M ionic strength. Panel b was adapted from Howard *et al.* [61] with permission from American Chemical Society.

Upon ligand-polyelectrolyte association (such as protein–DNA association), the condensed counterions can be released from the polyelectrolyte due to the formation of intermolecular ion pairs at the ligand–polyelectrolyte interface (Figure 7a). The release of condensed ions is entropically favorable, which is akin to the entropic effect of the release of the water molecules upon ion pair formations (Section 2.2). Considering this effect, Manning gave an analytical expression for the binding free energy as follows [59,60]:

(4) $$\Delta G=\Delta G_{0}-(Z-1)RT-ZRT\ln\{{\left[M^{+}\right]}_{cond}/[M^{+}]\}$$

where *Z* is the number of released counterions for each polyelectrolyte molecule upon ligand association and *R* is the gas constant. As described in Section 4.2, the entropic gain arising from the release of condensed counterions can be experimentally studied using measurements of the binding equilibrium constants at distinct concentrations of monovalent cations.

Counterion condensation theory may appear to be a crude approximation for the distribution of condensed ions and charges on linear polyelectrolyte. In fact, as displayed in Figure 7b, some computational studies of the condensed counterions around DNA [61,62,63,64,65,66,67] suggest that the actual spatial distribution of counterions significantly differs from the cylindrical uniform distribution around each charge assumed in the counterion condensation theory. However, the validity and usefulness of the counterion condensation theory have been proven for many protein–DNA associations through experimental studies [68,69,70,71,72] and also through computational studies based on the Poisson-Boltzmann equation [59,73,74].

## 3. Studies of Dynamics and Kinetics of Ion Pairs

Crystal structures typically show either a CIP or SIP state for each ion pair. However, the ion pairs of macromolecules should actually be dynamic in solution, given the relatively small free energy differences between the CIP and SIP states and the energy barriers between them. In this section, we review the kinetic and dynamic studies of the ion pairs.

### 3.1. Experimental Studies on the Ion-pair Dynamics of Small Compounds by Time-resolved Absorption Spectroscopy

**Figure 8:**
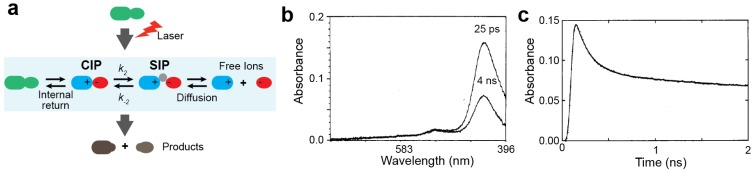
Kinetic studies of the CIP–SIP transitions by time-resolved absorbance spectroscopy. (**a**) Scheme for kinetic measurements of the laser-induced reactions involving CIP–SIP transitions. (**b**,**c**) Time-dependent absorption data for 10 μM diphenylmethyl chloride in acetonitrile. Panel b shows the absorption spectra at 25 ps and 4 ns after 266-nm irradiation. Panel c shows the time-course data of the absorbance at 440 nm. Panels b and c were adapted from Peters and Li [75] with permission from American Chemical Society.

Direct observation of the transitions between CIP and SIP states by experiment is challenging because the processes are very rapid. Despite this challenge, kinetic studies of the CIP–SIP transitions for some ion pairs of organic compounds in organic solvent were conducted as early as in the 1980s. The experimental approach in these studies is depicted in Figure 8a. They utilized time-resolved absorption spectroscopy together with a laser pulse that instantly (within ~10^−10^ s) initiates or perturbs the reactions involving the ion pairs. Following the laser irradiation, the time-course of the light absorbance is measured at a picosecond resolution, which monitors the reaction processes (examples of the data are shown in Figure 8b,c).

Peters and co-workers pioneered this approach for the kinetic studies of organic reactions involving CIP and SIP states [75,76,77,78,79]. They applied the time-resolved absorption spectroscopy for investigating transient ion pairs produced by the photoreduction of benzophenon by aromatic amines, such as diethylaniline and dimethylaniline, in ethanol and ethanol–acetonitrile mixtures [75,76,77,78]. Laser irradiation for 25–400 ps on the solution produces ion pairs of benzophenon radical anions and amino cations. Benzophenon radical anions exhibit different absorption spectra for the CIP and SIP states. Using the time-resolved absorption data, they determined the kinetic rate constants for the CIP–SIP transitions. They also demonstrated that the equilibrium between the CIP and SIP states could be shifted under different solution conditions. Later, they also studied the dynamics of the CIP–SIP exchange for the diphenylmethyl cation–chloride anion (DPM^+^Cl^−^) complex [75]. They demonstrated that the dynamic transitions between the CIP and SIP states occur on a sub-nanosecond timescale.

Using similar approaches, Kochi and co-workers conducted kinetic studies of ion pairs generated by the laser-induced charge-transfer excitation of the mixture of anthracene (or its derivatives) and tetranitromethane in a dichloromethane or acetonitrile solvent [17,18]. Laser irradiation of the anthracene–tetranitromethane mixture produced transient ion pairs of an arene cation and a trinitromethide anion. By time-resolved absorption spectroscopy, they investigated the kinetics and thermodynamics of the CIP–SIP exchange for this system in great detail. They found that the CIP–SIP transitions occur on a pico- to nanosecond timescale and that the free energy difference between the CIP and SIP states ranges from 1 to 2 kcal/mol.

Unfortunately, the applicability of the time-resolved absorption spectroscopy-based approach on the investigations of the ion-pair kinetics is very limited because this requires the activation by laser irradiation and the spectroscopic signatures of distinct states of ion pairs. To date, data on the ion-pair kinetics from this approach are available only for ion pairs involving aromatic moieties in polar organic solvents. Nonetheless, these studies in the 1980s and 1990s provided important insight into the kinetics and thermodynamics of the CIP–SIP exchange and their roles in some chemical reactions.

### 3.2. Experimental Studies on the Macromolecular Ion Pairs by NMR Spectroscopy

**Figure 9:**
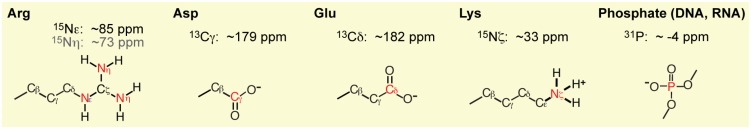
^13^C, ^15^N, and ^31^P nuclei useful for NMR investigations of ionic moieties of proteins and nucleic acids. Typical chemical shifts are also indicated.

NMR spectroscopy is a powerful tool for investigating the dynamics of biological macromolecules such as proteins, nucleic acids, and their complexes [80,81,82,83,84]. However, the vast majority of the NMR methods for the dynamics studies are for protein backbone NH or side-chain CH_3_ groups. Recently, several research groups developed NMR methods for investigating the dynamics of charged moieties of protein and nucleic acids. Figure 9 show the ^13^C, ^15^N, and ^31^P nuclei that are useful for such studies: namely, ^13^C nuclei of aspartate/glutamate carboxyl anions [85,86,87], ^15^N nuclei arginine guanidino cations [88,89,90,91,92,93,94,95,96,97], ^15^N nuclei of lysine amino cations [31,32,33,98,99,100,101,102,103,104,105,106,107,108,109]; and ^31^P nuclei of DNA and RNA phosphate anions [31,32,33,110,111,112]. NMR relaxation, three-bond scalar coupling, and hydrogen-bond scalar coupling data are particularly important for the investigations of ion pairs.

**Figure 10:**
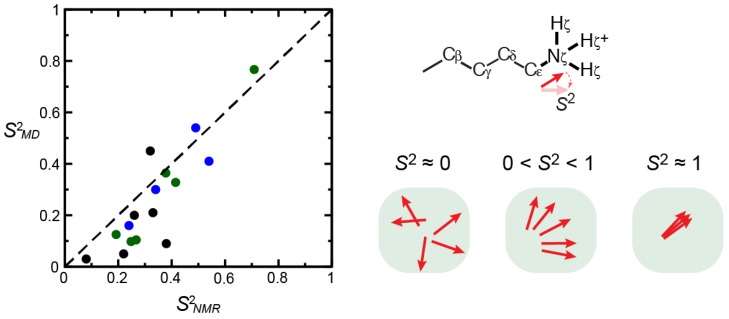
Order parameters *S*^2^ for C_ε_-N_ζ_ bond of Lys side-chain NH_3_^+^ groups. The graph shows correlation between *S*^2^ data from ^15^N NMR relaxation experiment and those calculated from MD trajectories for ubiquitin (green) [98], the Antp–DNA complex (blue) [32], and the Egr-1–DNA complex (black) [32].

The NMR relaxation-based methods can provide information on the mobility of ionic groups on a residue-specific basis. The relaxation data are often analyzed using the model-free formalism [113,114,115,116] with generalized order parameters and correlation times for bond reorientation. Order parameters *S*^2^ can provide a measure of the angular distribution of the particular bond vectors (e.g., C_ε_-N_ζ_ bond of Lys NH_3_^+^ groups). The *S*^2^ values range between 0 and 1; a smaller value represents a higher degree of mobility (*i.e.*, less ordered). Order parameters can also be calculated from MD simulations, which were found to be qualitatively consistent with the NMR-derived order parameters for charged side chains [32,87,94,98]. Some examples of *S*^2^ data for Lys NH_3_^+^ groups are shown in Figure 10. Charged moieties that form ion pairs generally exhibit larger order parameters than the corresponding moieties that do not form any ion pairs [31,32,86,87,94]. Nonetheless, many retain considerable mobility even in the ion pairs. For example, Lys NH_3_^+^ groups that directly interact with DNA phosphate groups exhibit substantial mobility, which should be entropically favorable for protein–DNA association [31,32,33].

**Figure 11:**
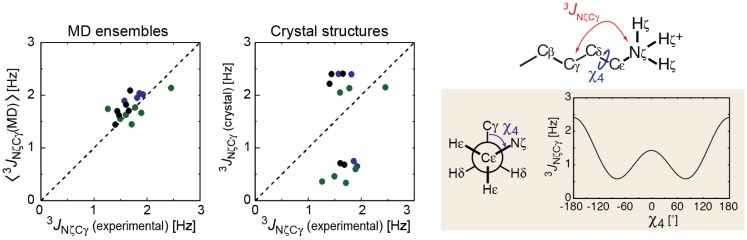
Three-bond scalar coupling constant ^3^*J*_NζCγ_ between Lys side-chain ^15^N_ζ_ and ^13^C_γ_ nuclei. Two correlations plots are shown for the experimental ^3^*J*_NζCγ_ data and those calculated from MD ensembles or crystal structures for ubiquitin (green) [106], the Antp–DNA complex (blue) [32], and the Egr-1–DNA complex (black) [32].

Three-bond scalar coupling constants are useful in detecting the dynamics of the bond torsion angles [106,117,118,119,120]. For investigations of ion-pair dynamics involving Lys side chains, three-bond scalar coupling between the ^15^N_ζ_ and ^13^C_γ_ nuclei (^3^*J*_NζCγ_) relevant to the *χ*_4_ torsion angle is particularly useful, because the ^15^N_ζ_ atoms are within the Lys NH_3_^+^ cations. Iwahara and co-workers measured the ^3^*J*_NζCγ_ coupling constants for lysine side chains and compared with those calculated from the structures [32,106] (Figure 11). Two correlation plots are displayed. One plot compares the experimental data with the ensemble averages of ^3^*J*_NζCγ_ coupling constants, <^3^*J*_NζCγ_>, calculated from the MD conformational ensemble, and the other plot compares with those calculated from single crystal structures. The MD ensemble <^3^*J*_NζCγ_> shows excellent agreement with the experimental data. In contrast, the ^3^*J*_NζCγ_ constants calculated from the single crystal structures exhibited bimodal distributions with two clusters corresponding to the *trans* and *gauche* *χ*_4_ conformers, and there values show poor agreement with the experimental NMR data. This remarkable difference between these plots suggests that the actual Lys *χ*_4_ torsion angles are as dynamics as observed in the MD simulations.

**Figure 12:**
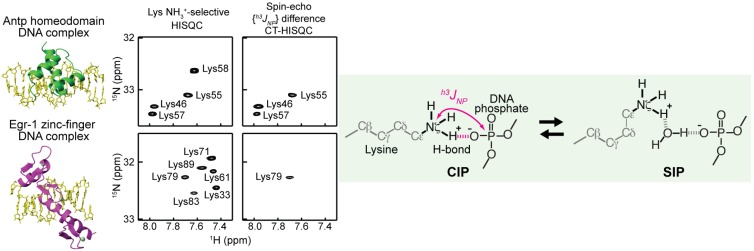
Detection of hydrogen-bond scalar coupling ^h3^*J*_NP_ between the Lys side-chain ^15^N and DNA phosphate ^31^P nuclei across a CIP. The coupling constants ^h3^*J*_NP_ can be measured with the pulse sequences of Anderson *et al.* [31]. The spin-echo ^h3^*J*_NP_ difference constant-time HISQC spectra give signals only when coupling is sizable [31]. Lys46, Lys55, Lys57 and Ly79 form intermolecular ion pairs with DNA phosphate groups, for which the CIP state is major [32].

Hydrogen-bond scalar coupling data are useful for detecting the CIP state of the ion pairs, and SIP cannot exhibit this type of coupling. Since late 1990s [121,122,123,124], hydrogen-bond scalar couplings have been observed for some different types of hydrogen bonds in proteins and nucleic acids (reviewed in Refs. [125,126]). Liu *et al.* detected hydrogen-bond scalar coupling between Arg71 ^15^N_ε_ and Asp100 ^13^C_γ_ of the FKBP12 protein, which indicates that the ion pair of these side chains is predominantly in the CIP state [127]. The measurement of the hydrogen-bond coupling is nontrivial because of its small magnitude (typically < 1 Hz). However, even for relatively large systems, such small hydrogen-bond scalar coupling constants can be measured for Lys side-chain NH_3_^+^ groups, owing to their very slow transverse ^15^N relaxation [31,32,106,128] (for example, see Figure 12). For ion pairs, the hydrogen-bond scalar coupling data provide direct evidence for the CIP states that involve hydrogen bonds. Iwahara and coworkers observed hydrogen-bond scalar coupling between the Lys side-chain ^15^N and DNA phosphate ^31^P nuclei in the HoxD9–DNA, Antp–DNA, and Egr-1–DNA complexes [31,32,33,109,128].

For Lys side chains, the rotations of NH_3_^+^ groups along C_ε_-N_ζ_ bonds can be analyzed using NMR relaxation data. It was found that the C_ε_-N_ζ_ bond rotations of Lys NH_3_^+^ groups occur on a 10^−12^–10^−10^ s timescale [31,32,98,108]. For NH_3_^+^ groups that form ion pairs with other moieties, the NH_3_^+^ bond rotations tend to be slower, but still occur on a pico- to nanosecond timescale. Zandarashvili and Iwahara recently studied energy barriers for NH_3_^+^ rotations for Lys side chains that form ion pairs with DNA phosphate groups by measuring the bond-rotation correlation time at some distinct temperatures [108]. Based on transition state theory, they analyzed the energy barriers for NH_3_^+^ rotations and compared to those for CH_3_ rotations. The enthalpies of activation for the NH_3_^+^ rotations were found to be significantly higher than those for the CH_3_ rotations, which can be attributed to the requirement of hydrogen bond breakage. However, the entropies of activation substantially reduced the overall free energies of activation for the NH_3_^+^ rotations to a level comparable to those for the CH_3_ rotations. The transient breakage of hydrogen bonds in the transition state for the NH_3_^+^ rotations could give more freedom to the water molecules, thereby increasing the entropy of the transition state. The reduction in energy barriers via the entropic mechanism might accelerate molecular processes requiring hydrogen bond breakage and play a kinetically important role in protein function [108].

### 3.3. Computational Studies on Dynamics of Macromolecular Ion Pairs

Because the free energy differences between the CIP and SIP states and the energy barriers between them are relatively small (Section 2.5), many ion pairs should undergo dynamic CIP–SIP equilibria. Some studies using MD simulations have shown the dynamic nature of the ion pairs of the protein side chains [129,130,131,132,133,134,135]. A direct evidence for such dynamic ion pairs is that the p*K*_a_ values predicted from the structure ensemble from MD or MC simulations are more accurate than those predicted from a single crystal structure, even for residues that are not affected by crystal packing [132,133,136,137,138,139,140]. Consideration of any single structure is obviously inadequate to describe behavior of ion pairs.

**Figure 13:**
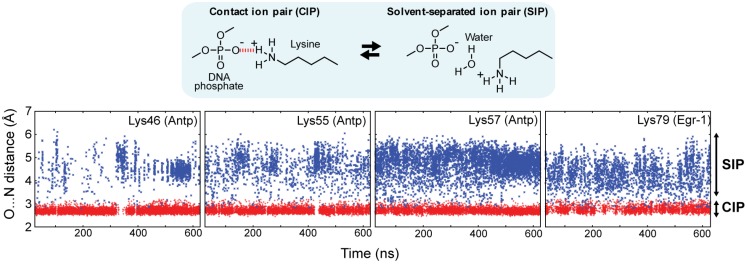
Dynamic transitions between the CIP (red) and SIP (blue) states of the intermolecular ion pairs of Lys side-chain NH_3_^+^ and DNA phosphate groups observed in the 0.6-μs MD simulations for the Antp–DNA and Egr-1–DNA complexes. Trajectories of distances from the Lys N_ζ_ atoms to the closest DNA phosphate oxygen atoms are shown for the intermolecular ion pairs for which the presence of CIP was experimentally confirmed. Adapted from Chen *et al.* [32].

Recently, using MD simulations, Chen *et al.* investigated CIP–SIP transitions for intermolecular ion pairs in the Antp homeodmain–DNA and Egr-1 zinc-finger–DNA complexes [32]. They monitored the contacts between each Lys side chain group and any DNA phosphate group in the 0.6-μs MD trajectories (Figure 13). For all of the Lys NH_3_^+^ groups that could directly contact DNA phosphate, the N…O distances dynamically fluctuated between two ranges: one between 2.5 and 3.2 Å, corresponding to the CIP states, and the other between 3.8 and 6.0 Å, corresponding to the SIP states. The transitions between the CIP and SIP states occurred on a pico- to nanosecond timescale. This observation was consistent with NMR relaxation and scalar coupling data [32], suggesting that the intermolecular ion pairs are as dynamic as seen in the MD simulations.

## 4. Experimental Studies of the Energetics of Ion Pairs in Biological Macromolecular Systems

For small ions, there are many experimental methods for the thermodynamic investigations of ion pairs such as dielectric relaxation spectroscopy, ultrasonic relaxation, and vibrational spectroscopy (e.g., reviewed in Reference [22]). Compared with the wealth of the methodologies that are available for small ion pairs, only a very limited number of experimental methods are available for the thermodynamic investigations of ion pairs of biological macromolecules. Here, we introduce some of these methods.

### 4.1. Experimental Analysis of the Energetics of Ion Pairs in Proteins

Because of the presence of multiple ion pairs of acidic and basic side chains, it is not trivial to analyze the energetics of particular ion pairs in proteins by experiment. The free energy of the interaction of a particular ion pair between two protein side chains is often analyzed using the double-mutant cycle method [141,142,143,144]. This method requires the analysis of four proteins: the wild-type protein with both side chains of the ion pair retained (X^+^Y^−^); two single-substitution mutants (X^+^N and NY^−^) with either of the side chains mutated to a neutral side chain (Ala in many cases); and a double-substitution mutant (NN) with both side chains mutated. The free energy, Δ*G*, for a particular molecular process of interest (e.g., protein folding) is measured for each of the four constructs. The coupling energy (ΔΔ*G*_int_) due to the ion-pair interaction between the two side chains X^+^ and Y^−^ is calculated from the four Δ*G* values as follows [141,142,143,144]:

(5) $$\Delta \Delta G_{\mathrm{int}}=\left\{\Delta G({\text{X}}^{\text{+}}{\text{Y}}^{\text{-}})-\Delta G({\text{NY}}^{\text{-}})\right\}-\left\{\Delta G({\text{X}}^{\text{+}}\text{N})-\Delta G(\text{NN})\right\}$$

**Figure 14:**
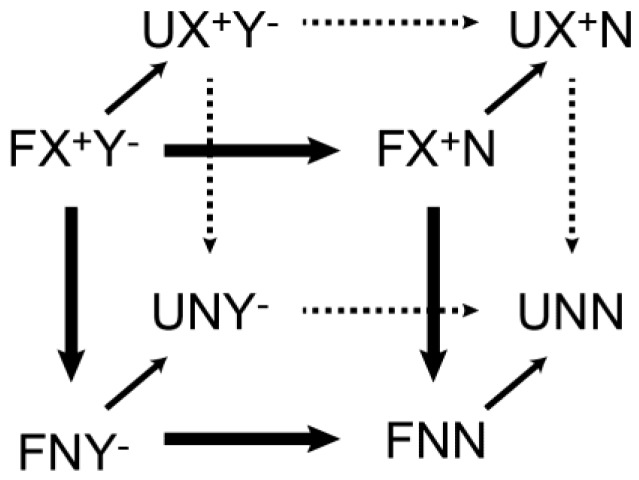
Double-mutant cycle to investigate the coupling energy for a particular ion pair of charged side chains X^+^ and Y^−^ in protein [142]. N represents a neutral side chain introduced by mutation. U and F represent the unfolded, and folded states, respectively.

This is equivalent to another common form:

(6) $$\Delta \Delta G_{\mathrm{int}}=\Delta \Delta G({\text{X}}^{\text{+}}{\text{Y}}^{-}\to \text{NN)}-\Delta \Delta G({\text{X}}^{\text{+}}{\text{Y}}^{-}\to {\text{X}}^{\text{+}}\text{N)}-\Delta \Delta G({\text{X}}^{\text{+}}{\text{Y}}^{-}\to {\text{NY}}^{-}\text{)}$$

where ΔΔ*G*(A → B) represents Δ*G*(B)-Δ*G*(A). When Δ*G* of protein folding is considered (Figure 14), the observed coupling energy is given by [142]:

(7) $$\Delta \Delta G_{\mathrm{int}}={\left(\Delta \Delta G_{\mathrm{int}}\right)}_{F}-{\left(\Delta \Delta G_{\mathrm{int}}\right)}_{U}$$

where (ΔΔ*G*_int_)_F_ and (ΔΔ*G*_int_)_U_ represent the ion-pair coupling energies for the folded and unfolded, respectively. Typically, (ΔΔ*G*_int_)_U_ = 0 is assumed because formation of X^+^-Y^−^ ion pair in the unfolded state seems unlikely. However, if this assumption is invalid, ΔΔ*G*_int_ = 0 does not necessarily imply that X^+^ and Y^−^ are not coupled; rather, it could imply that (ΔΔ*G*_int_)_F_ and (ΔΔ*G*_int_)_U_ are equal [143].

The coupling energy (ΔΔ*G*_int_) is related to the electrostatic and desolvation energies for the X^+^-Y^−^ ion pair. If CIP is the major state of the X^+^Y^−^ ion pair, the observed coupling energy is given by [141,144]:

(8) $$\Delta \Delta G_{\mathrm{int}}=\Delta G_{{\text{X}}^{\text{+}}{\text{Y}}^{\text{-}}\text{elec}}-\Delta \Delta G_{{\text{X}}^{\text{+}}\text{desolv}}-\Delta \Delta G_{{\text{Y}}^{\text{+}}\text{desolv}}$$

where Δ*G*_X_^+^_Y_^−^_elec_ represents to the electrostatic free energy for the X^+^-Y^−^ ion pair and ΔΔ*G*_disolv_ represents changes in the desolvation free energy upon mutation of counterion. If SIP is the major state for the X^+^-Y^−^ ion pair, the ΔΔ*G*_disolv_ terms could be negligible. Ionic-strength dependence data could allow for estimating the electrostatic and non-electrostatic components of ΔΔ*G*_int_ for an ion pair [144,145].

The double-mutant cycle method was used to assess stabilization of β-sheet [145,146,147] and α-helix [144] by ion pairs. These studies deduced propensities of amino-acid types and positions that would favor ion-pair formation stabilizing secondary structure. A majority of ion pairs at protein surface contribute to folding with typical stabilizing energies of ~0.3–1.5 kcal/mol. Cooperativity between ion pairs was also investigated by the double-mutant cycle methods [146,148]. The energetics of ion pairs provides a useful guideline for protein engineering [149,150].

### 4.2. Entropic Analysis of the Counterion Release upon Ion-Pair Formation between Protein and DNA

**Figure 15:**
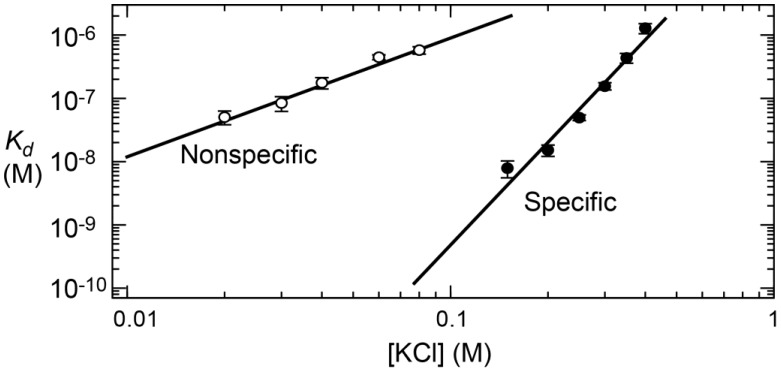
Log–log linear relationship between salt concentration and dissociation constant *K*_d_ (= 1/*K*_a_) for specific and nonspecific complexes of the Egr-1 zinc-finger protein and DNA [152]. Adapted from Ref. [145] with permission from Elsevier.

As described in Section 2.7, the release of condensed counterions upon the formation of protein–DNA complex renders an entropic gain. This entropic effect depends on the overall concentration of the cations in solution. Based on Manning’s counterion condensation theory, Record and co-workers developed an experimental method for investigations of the entropic effect arising from the counterion release upon DNA–ligand ion-pair formation [15,151]. According to this theory, the relationship between the equilibrium constant *K*_a_ = [DL]/([D][L]) for the DNA–protein interaction and monovalent cation concentration [*M*^+^] is given by:

(9) $$\text{log}K_{a}=\text{log}K_{a,1M}-z\varphi \text{log}[M^{+}]$$

where *z* represents the number of intermolecular ion pairs formed by DNA and the ligand; *φ*, a parameter reflecting the fraction of a counterion thermodynamically bound per phosphate of DNA in the free state; and *K*_a,1M_, the equilibrium constant at [*M*^+^] = 1 M (e.g., 1 M NaCl). The linear relationship between log[*M*^+^] and log*K*_a_ can easily be observed experimentally (some examples shown in Figure 15). From Equation (9), one can obtain:

(10) $$\Delta G_{obs}=\Delta G_{0}+z\psi RT\text{ln}[M^{+}]$$

The second term arises from the entropic change due to the release of condensed counterions from DNA upon the DNA–ligand complex formation. The entropic change from this polyelectrolyte effect for DNA is given by [153]:

(11) $$\Delta S_{PE}=-z\varphi R\text{ln}[M^{+}]$$

In these equations, the polyelectrolyte effect is considered for DNA, but not for protein. This is valid because the condition for counterion condensation (*i.e.*, *ζ* > 1; see Section 2.7) is satisfied for DNA but is unsatisfied for typical proteins. The entropic change Δ*S*_PE_ can be determined from the slope of the log–log linear relationship. Furthermore, the value of *φ* for B-form DNA is known to be 0.88, and one can also estimate *z*, the number of intermolecular ion pairs formed by protein and DNA. The validity of these analyses were experimentally confirmed for many protein–DNA complexes as reviewed in Reference [70].

## 5. Future Perspectives

Although recent advances in biochemical and biophysical research have substantially deepened our understanding of the ion pairs in biological systems, many issues remain to be addressed. We raise some of them here.

### 5.1. Potential Roles of CIP–SIP Transitions in Protein Functions

Roles of ion-pair dynamics in protein function have not been delineated yet, although it is not difficult to imagine that the motions of interacting ionized groups play important roles in macromolecular recognition, association, and enzymatic catalysis. The highly dynamic nature of the ion pairs could be kinetically advantageous for some molecular processes. Dynamic equilibria between the CIP and SIP states might be of particular importance. For example, in the target DNA search process where sequence-specific DNA-binding proteins locate particular sites, all CIPs with DNA need to be broken each time the protein moves from one nonspecific DNA site to another. Rapid CIP–SIP transitions should shorten the time necessary for breaking all CIPs and may facilitate the protein’s sliding on nonspecific DNA so that they can efficiently locate the target sites [31,154]. For many enzymes, ion pairs play a major role in the p*K*_a_ shift of the active-site side chains that are responsible for catalysis. Dynamic transitions between the CIP and SIP states might be important for some enzymatic reactions. In fact, the CIP–SIP transitions play a major role in solvolysis reactions of organic compounds in polar organic solvent [17,18,76,77,155]. Therefore, it is reasonable to speculate that CIP–SIP transitions play similar roles in enzymatic hydrolysis reactions. We expect that future research will reveal various roles of the CIP–SIP transitions in a variety of biomolecular processes.

### 5.2. Controversial Effects of Ion Pairs on Protein Stability

The role of side-chain ion pairs in protein stability remains controversial. Comparison of the thermophilic and nonthemophilic proteins clearly demonstrates the importance of surface side-chain ion pairs [156,157]. In contrast, the extensive Ala mutations of Staphylococcal nuclease, BPTI, and Arc repressor suggest relatively minor roles of side-chain ion pairs in protein folding [158,159,160]. There is a growing amount of evidence for both the stabilizing and destabilizing effects of ion pairs for protein folding [161]. Such seemingly contradictory effects could be related to ion pairs in non-native or denatured states of the proteins [162,163] (see Equation (7)). This could also be related to entropic effects of the side-chain motions. Characterizations of the ion pairs in various states of proteins are required at both the atomic and molecular levels.

### 5.3. Necessity of Methodological Development in Ion-Pair Research for Biomolecules

The current repertoire of experimental methods for thermodynamic, kinetic, and dynamic investigations of ion pairs in biological macromolecules remains relatively poor compared with those available for small ion pairs. Further methodological development is desired for the atomic-level investigations of biologically important ion pairs at molecular interfaces and enzymatic catalytic sites. NMR spectroscopy seems to be particularly promising in this regard. Further development is also desired for computational methods. For example, the root mean square difference (rmsd) between the p*K*_a_ values from experimental studies and those from the best computational methods are currently as high as 1 p*K*_a_ unit [43]. Because of the empirical nature of the force field parameters, validation of classical MD simulation data by experimental means is crucial, particularly for electrostatic issues such as ion pairs. In principle, this is not the case for *ab initio* MD. However, *ab initio* MD is computationally expensive and its applicability to ion pairs is currently limited to small systems and a short timescale (<100 ps) [164,165,166]. Improvement in computational speed may enable applications of *ab initio* MD to biological ion pairs over a longer timescale. With regard to classical MD, there was remarkable progress in the experiment-based improvement of force field parameters [167,168,169,170,171,172,173], but the optimized parameters were primarily for the protein backbone. We expect that future research on ion pairs will enable experiment-based validation and the improvement of force-field parameters that are relevant to the electrostatic interactions. Such validation and experiment-based improvement of the force field parameters can improve the *in silico* screening and design of drugs involving ion pairs.

## 6. Conclusions

In this review, we have introduced some fundamental concepts on physiochemical properties of ion pairs and given an overview of recent advances in research on biomolecular ion pairs. Although crystal structures of biomolecules typically show either CIP or SIP state for each ion pair, there are a growing number of evidences for the dynamic equilibria between the CIP and SIP states. This dynamic behavior observed for the ion pairs of biological molecules is reasonable in light of the fundamental concepts that were established for small ions over the last century. The ion-pair dynamics can be of functional importance for biological molecules such as proteins and nucleic acids. We expect that further advances in experimental and computational research on biological ion pairs will deepen our mechanistic understanding of various biomolecular processes and will also facilitate protein engineering and drug development.
